# Brainstem and striatal volume changes are detectable in under 1 year and predict motor decline in spinocerebellar ataxia type 1

**DOI:** 10.1093/braincomms/fcaa184

**Published:** 2020-12-15

**Authors:** Timothy R Koscik, Lauren Sloat, Ellen van der Plas, James M Joers, Dinesh K Deelchand, Christophe Lenglet, Gülin Öz, Peggy C Nopoulos

**Affiliations:** 1 Department of Psychiatry, University of Iowa Carver College of Medicine, Iowa City, IA 52242-1000, USA; 2 Department of Radiology, Center for Magnetic Resonance Research, University of Minnesota, Minneapolis, MN 55455, USA

**Keywords:** spinocerebellar ataxia, biomarkers, motor disorders, volumetry

## Abstract

Spinocerebellar ataxia type 1 is a progressive neurodegenerative, movement disorder. With potential therapies on the horizon, it is critical to identify biomarkers that (i) differentiate between unaffected and spinocerebellar ataxia Type 1-affected individuals; (ii) track disease progression; and (iii) are directly related to clinical changes of the patient. Magnetic resonance imaging of volumetric changes in the brain may be a suitable source of biomarkers for spinocerebellar ataxia Type 1. In a previous report on a longitudinal study of patients with spinocerebellar ataxia Type 1, we evaluated the volume and magnetic resonance spectroscopy measures of the cerebellum and pons, showing pontine volume and pontine *N*-acetylaspartate-to-*myo*-inositol ratio were sensitive to change over time. As a follow-up, the current study conducts a whole brain exploration of volumetric MRI measures with the aim to identify biomarkers for spinocerebellar ataxia Type 1 progression. We adapted a joint label fusion approach using multiple, automatically generated, morphologically matched atlases to label brain regions including cerebellar sub-regions. We adjusted regional volumes by total intracranial volume allowing for linear and power-law relationships. We then utilized Bonferroni corrected linear mixed effects models to (i) determine group differences in regional brain volume and (ii) identify change within affected patients only. We then evaluated the rate of change within each brain region to identify areas that changed most rapidly. Lastly, we used a penalized, linear mixed effects model to determine the strongest brain predictors of motor outcomes. Decrease in pontine volume and accelerating decrease in putamen volume: (i) reliably differentiated spinocerebellar ataxia Type 1-affected and -unaffected individuals; (ii) were observable in affected individuals without referencing an unaffected comparison group; (iii) were detectable within ∼6–9 months; and (iv) were associated with increased disease burden. In conclusion, volumetric change in the pons and putamen may provide powerful biomarkers to track disease progression in spinocerebellar ataxia Type 1. The methods employed here are readily translatable to current clinical settings, providing a framework for study and usage of volumetric neuroimaging biomarkers for clinical trials.

## Teaser

There is an urgent need for biomarkers that track neurodegenerative disease progression. Pontine and putamen volume decreases are specific to affected individuals, track disease progression within 6–9 months and are directly related to clinical changes in individuals affected by spinocerebellar ataxia Type 1, making them potential biomarkers for clinical trials.

## Graphical Abstract

**Graphical Abstract:**
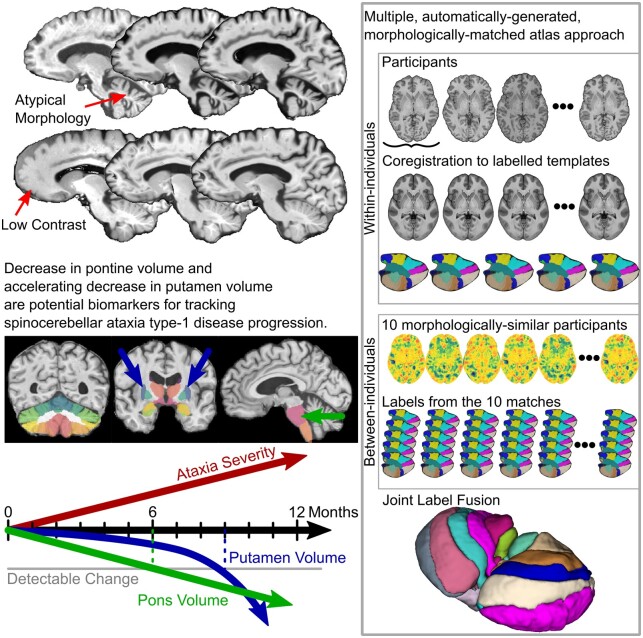

## Introduction

Spinocerebellar ataxias (SCAs) are a set of autosomal dominantly inherited movement disorders for which there currently are no effective treatments. SCAs are a heterogenous family of ∼40 disorders with progressive loss of control of movement. Spinocerebellar ataxia Type 1 (SCA1) is caused by polyglutamine expansion of the *ataxin-1* gene (Orr *et al.*, 1993; Banfi *et al.*, 1994). As therapeutics for SCAs, including SCA1, are being developed, identification of specific and sensitive, non-invasive biomarkers of disease progression is critical.

Biomarkers that are useful for tracking the efficacy of treatments must meet several criteria: (i) potential biomarkers should differentiate individuals affected by the disease relative to those unaffected by the disease. (ii) Changes in the biomarker must be reliably detectable within affected individuals, in addition to detectable between affected and unaffected individuals. (iii) Biomarker changes must occur within a timeframe that will allow monitoring treatment efficacy; slower rates of change are more difficult to track and evaluate. (iv) Changes in biomarkers must predict disease progression; conversely, lack of change in a sensitive biomarker would indicate lack of disease progression. Biomarkers are not necessarily sufficient as diagnostic criteria for a disease, rather they are useful for track progression in diagnosed individuals. In addition, biomarkers are best when they can be measured non-invasively and consistently in standard clinical settings. In the case of SCA1, neuroimaging biomarkers show particular promise (for a recent systematic review, see Öz *et al.*, 2020).

In 2013, Reetz *et al.* published on the findings from the EUROSCA consortium evaluating change over time in patients with SCA1, spinocerebellar ataxia Type 3 (SCA3) and spinocerebellar ataxia Type 6 (SCA6) over a 2-year interval. For SCA1, they reported changes in brainstem, pons, caudate and putamen, but no substantial change in cerebellar volume. Results from the BIOSCA study suggest that volumetric changes in pons and cerebellum in SCA1, SCA3 and SCA6, track disease progression better than clinical scores (Adanyeguh *et al.*, 2018). Importantly, at baseline, the brainstem and cerebellar volumes were already substantially below normal in this ataxic cohort while the caudate and putamen volumes were normal at baseline (Reetz *et al.*, 2013). This suggests that brainstem and cerebellum changes occur early in the course of disease [and likely in the pre-manifest phase (Maas *et al.*, 2015)], whereas regions such as the striatum degenerate later in disease. It also suggests a ‘floor effect’ for change in the cerebellum meaning that if the volume is already so low at baseline, there may not be substantial room for significant change over time, whereas the striatum may be changing more rapidly given the normal volume at baseline.

Evaluation of brain regions that are considered to be part of an integrated circuit is vital in understanding how regions may degenerate over time, or even how other regions in that circuit may play a compensatory role for other nodes of the circuit that are degenerating. The cerebellum is a critical node in motor control networks that involve integration with pontine nuclei and the striatum (Hoshi *et al.*, 2005; Bostan *et al.*, 2010, 2013). Evaluating changes in brain regions over time that are associated with cerebellar–striatal circuitry could not only help identify the most sensitive markers of change, it could also highlight the need to evaluate functional circuitry in SCA1 as well.

In the era of clinical trials aimed at slowing disease progression, showing any changes over a period of time that a trial can accommodate would be of great advantage. We previously reported longitudinal volumetric and neurochemical changes in the cerebellum and pons in patients with SCA1 (Deelchand *et al.*, 2019). In a sample of early-to-moderate stage patients scanned up to three times with 1.5-year intervals, we found pontine total *N*-acetylaspartate-to-*myo*-inositol ratio and pontine volume to be the most sensitive magnetic resonance (MR) measures to change. That report was focussed on limited brain regions as the emphasis was on the neurochemical changes.

As a follow-up analysis, we utilized this same patient sample, expanding the volumetric assessment to whole brain using an advanced labelling technique. The methodologic advances of this analysis over prior studies includes (i) use of a high-field, 3T magnet; (ii) statistical modelling of time, which allows for earliest detection of change; and (iii) a whole brain approach utilizing advanced labelling and quantification of volumes. These methodological advances may help to better identify those regions that change the most quickly and are predictive of motor changes, making them candidates for sensitive biomarkers of disease progression.

## Materials and methods

### Participants and study design

We utilized recently reported data (Deelchand *et al.*, 2019) and conducted additional volumetric analyses. Briefly, 16 genetically confirmed individuals with SCA1 (nine women and seven men) and 21 matched unaffected participants (8 women and 13 men) gave informed consent approved by the Institutional Review Board at the University of Minnesota in accordance with the Declaration of Helsinki and participated in the study. SCA1-affected individuals were recruited as an early-stage cohort [Scale for the Assessment and Rating of Ataxia (SARA) of <15] as the likely candidates for future clinical trials. Participants were evaluated at intake and followed up at ∼18 and 36 months (three visits) between March 2011 and June 2015. Twenty-one unaffected individuals and all SCA1-affected individuals returned at 18 months. Thirteen SCA1-affected individuals returned at 36 months, while three declined to return due to disease progression and travel difficulty. To match the SCA1-affected cohort, only 15 unaffected individuals were invited for their 36-month visit. At each visit, participants underwent MR imaging and were assessed using the SARA, which evaluates eight quantitative features for gait, stance, sitting, speech disturbance and limb kinetic functions (Schmitz-Hübsch *et al.*, 2006), where scores range from 0 (no ataxia) to 40 (most severe ataxia). We excluded one unaffected individual and three SCA1-affected individuals from our analyses due to excessive noise and/or motion during one of their scans that precluded accurate volumetric labelling; our final sample consisted of 13 SCA1-affected (eight women, five men) and 20 unaffected individuals (8 women, 12 men).

### MR acquisition

MR images were acquired on a 3T whole-body Siemens MR scanner (Siemens Medical Solutions, Erlangen, Germany), where the standard body coil was used for excitation and a 32-channel receive-only head coil was used for signal reception. At each visit, 3D T_1_-weighted MPRAGE images were acquired with the following parameters: 1-mm^3^ isotropic resolution, repetition time = 2530 ms, echo time = 3.65 ms, inversion time = 1100 ms, flip angle = 7° and GRAPPA acceleration factor = 2.

### MR processing and volumetrics

Advanced Normalization Tools (Avants *et al.*, 2009), FSL [FMRIB (the Functional Magnetic Resonance Imaging of the Brain) Software Library] (Jenkinson *et al.*, 2012), as well as custom scripts were used for all MR processing, volumetric segmentations and analyses (all custom scripts will be made available online). First, T_1_-weighted images were rigidly aligned to a common template to approximate anterior commissure, posterior commissure alignment. Next, images were denoised with an adaptive non-local means approach using a Rician noise model (Manjón *et al.*, 2010). Next, preliminary brain extraction was performed using Advanced Normalization Tools brain extraction (Tustison *et al.*, 2014), then iterative inhomogeneity correction–tissue segmentation was performed using Advanced Normalization Tools atropos (Avants *et al.*, 2011) and the N4 debiasing algorithm (Tustison *et al.*, 2010). After debiasing, brain extraction was repeated to generate a more accurate brain mask.

There are several methodological advancements in regional labelling procedures that would benefit measurement of neuroimaging biomarkers. Multi-atlas methods outperform single-atlas deformations in both accuracy and reliability of segmentations (Aljabar *et al.*, 2009; Pierson *et al.*, 2011; Chakravarty *et al.*, 2013; Wang *et al.*, 2013). Cleaned images were labelled using a multiple, automatically generated, morphologically matched atlas approach (MAGMA). MAGMA combines several existing methods and optimizations that produce regional labels that are relatively invariant to site, scanner, morphological abnormalities and scan quality. Non-cerebellar regions and cerebellar regions were labelled separately in order to optimize registration parameters and computations to each sub-region. The MAGMA procedure was adapted from the BRAINSAutoWorkup pipeline, which optimizes tissue classification through an iterative framework and produces robust parcellation of brain regions (Pierson *et al.*, 2011). Briefly, brain regions were labelled using a multi-atlas, similarity-weighted, majority-vote procedure [joint label fusion (Wang *et al.*, 2013)] using a set of expert-segmented templates. For non-cerebellar regions, templates were adapted from the Desikan–Killiany atlas (Desikan *et al.*, 2006) and, for cerebellar sub-regions, the COBRAlab cerebellar templates and segmentations were used (Park *et al.*, 2014). To further improve parcellation accuracy for cerebellar parcellations, the MAGMA procedure implements a secondary procedure adapted from the MAGeT parcellation pipeline (Chakravarty *et al.*, 2013; Park *et al.*, 2014; Pipitone *et al.*, 2014), which creates a set of subject-to-subject registrations leveraging individual variation in neuroanatomy to improve label accuracy. In contrast to MAGeT, which pre-selects a sub-sample of exemplar images for use of all subject-to-subject registrations, MAGMA optimizes exemplar selection for each individual based on morphological similarity to the target image, further improving labelling accuracy. Briefly, each subject is normalized to a template space [an unbiased average T_1_-weighted image from the S1200 Human Connectome release (Van Essen *et al.*, 2013)], and Jacobian determinants of the deformation matrix to this space are calculated. MAGMA then selects a unique subset of exemplars from the entire sample (in this case 10 unique individuals) where their similarity in Jacobian determinants within the brain, as measured by Pearson correlation, is greatest. These subsets were generated uniquely for each subject and time point. The MAGMA procedure then performs a joint label fusion using this set of 10 exemplars each with five sets of labels from the cerebellar atlases (50 labelled images total) to generate the final, MAGMA labels for each individual. Finally, manual check for accuracy of regional label and editing was completed by an individual blind to the participant’s diagnosis.

**Figure 1:**
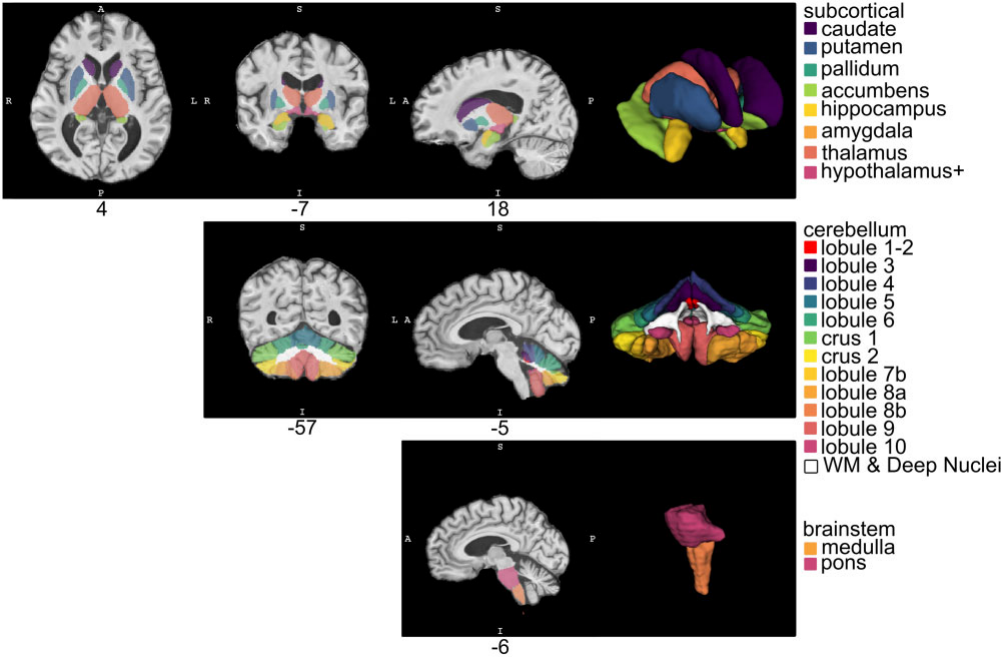
**Volumes of interest. Example of VOI labels in a SCA1 participant.** The top row depicts subcortical VOIs, including caudate, putamen, pallidum, nucleus accumbens, hippocampus, amygdala, thalamus and hypothalamus (including a portion of the midbrain), generated using BRAINSAutoWorkup. The middle row depicts cerebellar sub-region parcellations, including major lobules, generated using the MAGMA procedure. The bottom row depicts pons and medulla generated using FreeSurfer

We explored 32 volumes of interest (VOIs). Five were global measures of whole brain, cerebrum, cerebral grey matter, cerebral white matter (WM) and whole cerebellum volumes. The remaining 27 were sub-regions including the following (and shown in Fig. 1): cerebellar Lobule 1 and 2, cerebellar Lobule 3, cerebellar Lobule 4, cerebellar Lobule 5, cerebellar Lobule 6, cerebellum Crus 1, cerebellum Crus 2, cerebellar Lobule 7b, cerebellar Lobule 8a, cerebellar Lobule 8b, cerebellar Lobule 9, cerebellar Lobule 10, cerebellar WM and deep nuclei, corpus callosum, frontal lobe, occipital lobe, parietal lobe, temporal lobe, caudate, putamen, pallidum, accumbens, hippocampus, amygdala, thalamus, hypothalamus+ (includes some anterior midbrain in addition to hypothalamus proper) and brainstem.

### Statistical analysis

Much of the research on SCAs has used *estimated* total intracranial volume (ICV) from either FreeSurfer (Dale *et al.*, 1999) or subsampling methods (Eritaia *et al.*, 2000) to normalize for differences in brain size. These estimates have the potential to be inaccurate, which can inject estimated total ICV error into the normalized variables. While more difficult, labelling of intracranial voxels using multi-atlas methods provides a direct measurement of ICV.

First, regional brain volumes (VOIs) depend to some degree on the overall size of an individual, their heads and their brains. While some decrease in brain volume may be expected in a neurodegenerative disorder like SCA1, most observed differences in adult ICV likely reflect developmental differences since ICV reflects maximal brain growth rather than current brain volume. We are interested in change over time in VOIs (especially regional neurodegeneration), rather than differences in developmental processes between SCA1-affected and -unaffected adults. Thus, we adjusted VOIs by ICV using the power proportion method (Liu *et al.*, 2014), which allows for power-law relationships between ICV and VOIs in addition to linear or proportional relationships. Briefly, for each VOI, we fit a non-linear regression model,

$$\mathrm{VOI}=\alpha {\mathrm{ICV}}^{\beta },$$

where *α* is a constant and *β* is the scaling exponent of the power function (implemented in R, https://github.com/tkoscik/tkmisc, 4 November 2020, date last accessed). Once *β* is estimated for each regional volume, we divide each VOI by ICV raised to power *β*.

$${\mathrm{VOI}}_{\mathrm{adjusted}}=\frac{\mathrm{VOI}}{{\mathrm{ICV}}^{\beta }}.$$

In order to recover natural units, we scaled VOIs by centring on the adjusted mean and scaling by the adjusted standard deviation, then scaling to the unadjusted standard deviation and adding in the unadjusted mean.

$${\mathrm{VOI}}_{\mathrm{adjusted}}=\left(\frac{{\mathrm{VOI}}_{\mathrm{adjusted}}-\mu_{{\mathrm{VOI}}_{\mathrm{adjusted}}}}{\sigma_{{\mathrm{VOI}}_{\mathrm{adjusted}}}}\right)\times \sigma_{\mathrm{VOI}}+\mu_{\mathrm{VOI}}.$$

Second, since we are interested in change over time, we calculated change in VOIs from baseline. In addition, we decomposed age at testing into its variance subcomponents in order to model these effects independently, specifically baseline age (i.e. between-individuals variance) and change in age between visits (i.e. within-individuals variance).

A baseline analysis of volume differences was conducted comparing volumes of all regions across groups, using linear regression models that included age at baseline and gender as covariates. For each model, dependent variables (VOIs) were scaled such that beta coefficients in regression models were standardized to allow for comparison between VOIs, despite gross differences in regional volumes.

To address our first criterion for biomarkers—to differentiate individuals affected by SCA1 relative to those unaffected—we explored whether changes in VOIs within-individuals differs between SCA1-affected and -unaffected individuals. Changes should be disease related, not due to normal, age-related change; this is not a diagnostic tool where SCA1 is defined by molecular genetics, rather a means to differentiate abnormal from normal change. In other words, we evaluated interaction effects between groups (SCA1 affected versus unaffected) and elapsed time between visits. We used linear mixed effects (LME) models to predict changes in VOIs with fixed effects of group, elapsed time between visits, age at baseline and gender, as well as the critical interaction between group and elapsed time. We included individuals as random effects to account for repeated observations. This model was applied to each VOI separately and we controlled family-wise error rates using Bonferroni correction.

To address our second criterion for biomarkers—changes in the biomarker must be detectable and reliable within affected individuals—we explored whether changes in VOIs are observable within affected individuals only. This criterion is critical; SCA1-affected individuals will not enter a clinical setting with a matched comparison group; thus, successful biomarkers must track affected individuals, relative to their own baseline. We repeated LME models predicting changes in VOIs within SCA1-affected individuals only. We included fixed effects of elapsed time (the variable of interest), baseline age and gender, as well as the random effect of repeated measures within-participants in each of our models. Again, we applied Bonferroni correction to control the family-wise error rate.

To address our third criterion for biomarkers—changes must occur within a reasonable timeframe to monitor treatment efficacy—we explored when changes in regional volume exhibit detectable differences from zero within SCA1-affected individuals. We estimated the 99% confidence interval for the elapsed time main effect from our LME models. Once this confidence interval no longer overlapped zero (no detectable change), our models would suggest that we could detect a change within individuals with a high level of confidence. The faster a change can be detected within SCA1-affected regional volumes, the better for a potential biomarker.

To address our fourth criterion for biomarkers—changes in biomarkers must predict disease progression—we assessed which variables predict changes in disease progression. We use SARA scores as a measure of disease progression in SCA1, where increases in values indicate a greater disease burden. To capture change in disease burden, we subtracted baseline SARA scores from scores obtained at each follow-up visit. Given that rates of change between brain regions exhibit at least some multicollinearity (i.e. they all tend to decrease in SCA1) and there is the potential for over-fitting our data, we performed an LME-least absolute shrinkage and selection operator procedure, where predictors (change in regional brain volume) are penalized if they are less important in the model. This model allows for feature selection or demonstrating which predictors best explain the rate of change in SARA score. This method also includes a cross-validation procedure to select the penalization parameters that minimize prediction error. Our LME model that predicted change in SARA score includes: fixed effects for all VOIs that significantly differentiate groups and have detectable change within SCA1-affected individuals only, interaction effects between these VOIs and elapsed time, as well as main effects of elapsed time age at baseline and gender and random effects of participant.

### Data availability

Data and software can be made available upon reasonable request.

## Results

### Cohort characteristics

SCA1-affected and -unaffected individuals did not differ in age at baseline [*t*(30.9) = −0.191, *P* = 0.8499, mean SCA1 = 53.3 years, mean unaffected = 52.5 years], in elapsed time between first and second visit [*t*(24.3) = 0.867, *P* = 0.394, mean SCA1 = 1.6 years, mean unaffected = 1.5 years], nor in elapsed time between first and third visit [*t*(22.8) = 1.191, *P* = 0.246, mean SCA1 = 3.008 years, mean unaffected = 2.942 years]. Given that there were slightly more men in our unaffected group relative to the SCA1-affected group, we ran a chi-squared test, which suggests that the differences in gender distribution between groups were not significant [*χ*^2^(1) = 0.405, *P* = 0.524].

### Intracranial volume

SCA1-affected individuals had comparable mean ICV to unaffected individuals [*β* = −57 058.5, *t*(30.99) = −1.219, *P* = 0.232]. Both groups exhibited significant variance in ICV [SCA1 ICV (mm^3^): mean = 1 472 062.4, SD = 116 358.8, min = 1 288 409, max = 1 699 525; unaffected ICV (mm^3^): mean = 1 535 453.8, SD = 135 385.6, min = 1 292 165, max = 1 743 620]. Moreover, ICV was highly correlated with each VOI, with coefficients ranging from *r *=* *0.219 to 0.949 (see Supplementary Table 1). Correcting for this relationship, power parameters varied around 1 (relatively linear relationship) for cortical regions, but tended to be <1 for non-cortical regions (indicating that these regions scale at a lower rate with increasing ICV; for a listing of all values, see Supplementary Table 1). Critically, following power proportion corrections, virtually no correlation between VOIs and ICV remains, ranging from *r* = −0.0375 to 0.0234.

### Baseline volume differences

**Figure 2:**
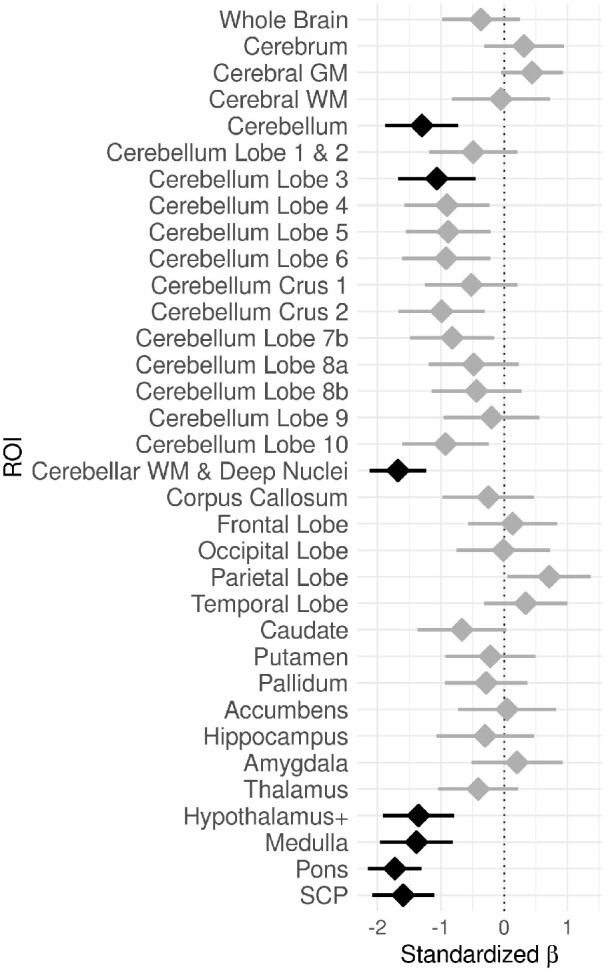
**Baseline volume differences.** Differences in volume between groups (SCA1 versus unaffected) at baseline. Values on the *x*-axis correspond to standardized beta coefficients from linear regression models, where negative values indicate lower volumes in SCA1 relative to unaffected individuals. A standardized beta of 1 indicates 1 standard deviation difference. Black indicates a significant group difference in the VOI after Bonferroni correction; grey indicates non-significant results. Error bars indicate 95% confidence intervals for standardized beta coefficients. The dotted vertical line indicates no difference between groups

At the time of initial assessment, the following regions were substantially lower in volume in the SCA1 patients compared to unaffected individuals: total cerebellar volume [*β* = −1.30, *t*(29) = −4.603, *P* = 0.00260], cerebellar Lobule 3 [*β* = −1.064, *t*(29) = −3.533, *P* = 0.0475] and cerebellar WM and deep nuclei [*β* = −1.678, *t*(29) = −77.644, *P* = 6.76 × 10^−7^] (all *P*’s Bonferroni corrected). All other cerebellar regions had lower baseline volumes, contributing to the aggregate total cerebellum volume; they were not significant after Bonferroni correction individually. Also lower in volume at baseline was the hypothalamus (VOI includes portions of the midbrain) [*β* = −1.353, *t*(29) = −4.939, *P* = 0.00102], medulla [*β* = −1.385, *t*(29) = −1.385, *P* = 0.00106], pons [*β* = −1.726, *t*(29) = −8.325, *P* = 1.205 × 10^−7^] and the superior cerebellar peduncle [*β* = −1.596, *t*(29) = −6.645, *P* = 9.38 × 10^−6^]. See Fig. 2 for visual display of the baseline volume analysis.

### Differences in VOI change between groups

**Figure 3:**
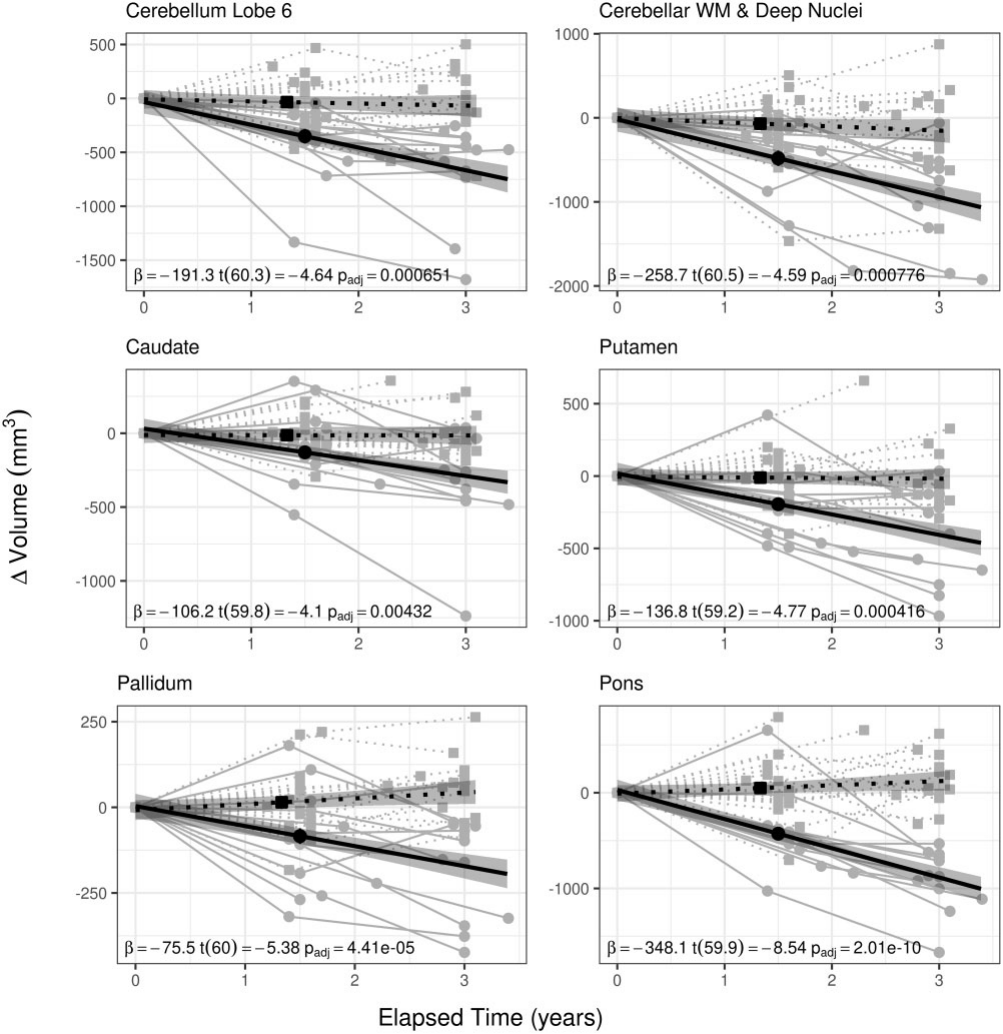
**Group by elapsed time interactions by VOI.** Regional brain volumes where volumetric change was predicted by a group (SCA1 affected versus unaffected) by elapsed time (time between visits) interaction. While most brain regions do not change in volume in unaffected individuals, volumes of cerebellar Lobule 6, cerebellar WM and deep nuclei, caudate, putamen, pallidum and pons decrease over time in SCA1-affected individuals. Solid lines indicate values for SCA1-affected individuals, where grey lines and circles represent observed values, and the black line represents the fitted model. Dotted lines indicate values for unaffected individuals, where grey lines and squares represent observed values and the black; dotted line is the fitted model. Shaded regions indicate 95% confidence intervals

Changes in VOIs exhibited different relationships between groups in relation to elapsed time. Several VOIs decreased at a faster rate in SCA1-affected individuals relative to unaffected individuals. LME models revealed several VOIs where change was predicted by the group by elapsed time interaction (all *P*’s are Bonferroni corrected). These include: cerebellar Lobule 6 [*β* = −191.258, *t*(60.3) = −4.64, *P* = 6.51 × 10^−4^], cerebellar WM and deep nuclei [*β* = −258.66, *t*(60.5) = −4.59, *P* = 7.76 × 10^−4^], caudate [*β* = −106.223, *t*(59.8) = −4.1, *P* = 0.00432], putamen [*β* = −136.797, *t*(59.2) = −4.77, *P* = 0.000416], pallidum [*β* = −75.485, *t*(60) = −5.38, *P* = 4.41 × 10^−5^] and pons [*β* = −348.144, *t*(59.9) = −8.54, *P* = 2.01 × 10^−10^] (Fig. 3). These regions meet our first criterion for a potential biomarker for SCA1 disease progression—the rate of change in cerebellar Lobule 6, cerebellar WM and deep nuclei, caudate, putamen, pallidum and pons differentiate between SCA1-affected and -unaffected individuals. For group by elapsed time interaction effects for all VOIs, see Table 1; for full LME model results, see Supplementary Table 2.

**Table 1:** Group by elapsed time interactions by VOI

VOI	β	SE	df	*t*	*P*	*P*_Bonferroni_
Whole brain	−8417.702	6623.368	60.3	−1.27	0.209	1
Cerebrum	−6864.891	6402.336	60.3	−1.07	0.288	1
Cerebral GM	−5325.858	3680.643	59.9	−1.45	0.153	1
Cerebral WM	82.611	2117.327	60.7	0.039	0.969	1
Cerebellum	−1111.813	337.35	60	−3.3	0.00165	0.0562
Cerebellar Lobules 1 and 2	6.182	2.126	60.3	2.91	0.00508	0.173
Cerebellar Lobule 3	−3.674	10.721	60.2	−0.343	0.733	1
Cerebellar Lobule 4	−14.073	19.859	60.4	−0.709	0.481	1
Cerebellar Lobule 5	−104.775	40.724	58.8	−2.57	0.0126	0.43
Cerebellar Lobule 6	−191.258	41.204	60.3	−4.64	1.91 × 10^−5^	6.51 × 10^−4^
Cerebellum Crus 1	−212.965	121.987	60.4	−1.75	0.0859	1
Cerebellum Crus 2	−397.944	197.379	61.9	−2.02	0.0481	1
Cerebellar Lobule 7b	−5.103	106.154	61	−0.0481	0.962	1
Cerebellar Lobule 8a	154.298	91.619	60.9	1.68	0.0973	1
Cerebellar Lobule 8b	57.53	61.447	60.4	0.936	0.353	1
Cerebellar Lobule 9	−45.321	41.75	59.4	−1.09	0.282	1
Cerebellar Lobule 10	28.049	17.78	59.8	1.58	0.12	1
Cerebellar WM and deep nuclei	−258.66	56.339	60.5	−4.59	2.28 × 10^−5^	7.76 × 10^−4^
Corpus callosum	−26.852	15.458	60.2	−1.74	0.0875	1
Frontal lobe	−5563.115	2729.405	60.1	−2.04	0.0459	1
Occipital lobe	521.456	740.068	60	0.705	0.484	1
Parietal lobe	−480.415	1743.822	59.8	−0.275	0.784	1
Temporal lobe	1107.655	1093.294	60.1	1.01	0.315	1
Caudate	−106.223	25.915	59.8	−4.1	0.000127	0.00432
Putamen	−136.797	28.651	59.2	−4.77	1.22E-05	0.000416
Pallidum	−75.485	14.032	60	−5.38	1.3E-06	4.41E-05
Accumbens	12.328	31.652	60.4	0.39	0.698	1
Hippocampus	−4.378	11.367	58.8	−0.385	0.702	1
Amygdala	−8.45	7.248	59.8	−1.17	0.248	1
Thalamus	−59.833	49.289	58.8	−1.21	0.23	1
Hypothalamus+	−78.27	41.578	60.5	−1.88	0.0646	1
Medulla	−29.044	58.287	59.9	−0.498	0.62	1
Pons	−348.144	40.743	59.9	−8.54	5.91E-12	2.01E-10
SCP	−4.505	2.773	60.8	−1.62	0.109	1

GM, grey matter.

### VOI change within SCA1-affected individuals

In line with our second criterion for a potential biomarker, change in several VOIs was observable over time within SCA1-affected individuals, without reference to the unaffected group. LME models revealed that elapsed time predicts change in cerebellar Lobule 6 [*β* = −210.984, *t*(24.8) = −6.03, *P* = 9.33 × 10^−5^], cerebellar WM and deep nuclei [*β* = −308.859, *t*(24.7) = −6.71, *P* = 1.76 × 10^−5^], corpus callosum [*β* = −46.005, *t*(24.6) = −3.96, *P* = 0.0193], caudate [*β* = −106.709, *t*(24.8) = −4.17, *P* = 0.0109], putamen [*β* = −141.078, *t*(24.7) = −5.68, *P* = 0.000229], pallidum [*β* = −58.671, *t*(24.6) = −4.49, *P* = 0.0049], thalamus [*β* = −210.56, *t*(24.9) = −6.36, *P* = 4.07 × 10^−5^] and pons [*β* = −304.985, *t*(25.1) = 9.8, *P* = 1.6 × 10^−8^]. For elapsed time effects for all VOIs, see Table 2; for full LME model results, see Supplementary Table 3.

**Table 2:** VOI change in SCA1-affected only

VOI	β	SE	df	*t*	*P*	*P*_Bonferroni_
Whole brain	−12 584.624	6057.012	24.7	−2.08	0.0483	1
Cerebrum	−10 848.905	5855.834	24.7	−1.85	0.0759	1
Cerebral GM	−6523.224	3313.096	24.7	−1.97	0.0603	1
Cerebral WM	−1610.601	1915.169	24.7	−0.841	0.408	1
Cerebellum	−971.808	305.995	24.6	−3.18	0.004	0.136
Cerebellar Lobules 1 and 2	1.645	1.275	24.7	1.29	0.209	1
Cerebellar Lobule 3	5.003	7.273	24.7	0.688	0.498	1
Cerebellar Lobule 4	−26.158	12.249	24.3	−2.14	0.043	1
Cerebellar Lobule 5	−40.215	31.987	23.1	−1.26	0.221	1
Cerebellar Lobule 6	−210.984	34.977	24.8	−6.03	2.75E-06	9.33E-05
Cerebellum Crus 1	−129.373	100.688	24.4	−1.28	0.211	1
Cerebellum Crus 2	−193.729	172.791	24.8	−1.12	0.273	1
Cerebellar Lobule 7b	11.347	69.978	24.7	0.162	0.873	1
Cerebellar Lobule 8a	19.061	86.51	24.6	0.22	0.827	1
Cerebellar Lobule 8b	−18.594	46.305	24.8	−0.402	0.691	1
Cerebellar Lobule 9	−5.616	38.073	24.1	−0.148	0.884	1
Cerebellar Lobule 10	31.742	14.051	25	2.26	0.0328	1
Cerebellar WM and deep nuclei	−308.859	46.01	24.7	−6.71	5.18E-07	1.76E-05
Corpus callosum	−46.005	11.63	24.6	−3.96	0.000569	0.0193
Frontal lobe	−5454.899	1638.349	24.9	−3.33	0.00271	0.0921
Occipital lobe	−284.578	708.166	24.6	−0.402	0.691	1
Parietal lobe	−2305.137	1287.966	24.7	−1.79	0.0858	1
Temporal lobe	1395.581	991.918	24.6	1.41	0.172	1
Caudate	−106.709	25.568	24.8	−4.17	0.000321	0.0109
Putamen	−141.078	24.83	24.7	−5.68	6.75E-06	0.000229
Pallidum	−58.671	13.059	24.6	−4.49	0.000144	0.0049
Accumbens	3.968	29.17	24.7	0.136	0.893	1
Hippocampus	1.551	9.005	25	0.172	0.865	1
Amygdala	−9.143	7.016	24.8	−1.3	0.204	1
Thalamus	−210.56	33.103	24.9	−6.36	1.2E-06	4.07E-05
Hypothalamus+	−89.29	30.356	24.7	−2.94	0.00699	0.238
Medulla	11.577	28.214	24.3	0.41	0.685	1
Pons	−304.985	31.126	25.1	−9.8	4.70E-10	1.60E-08
SCP	−8.247	2.218	24.9	−3.72	0.00102	0.0348

GM, grey matter.

### Timeframe for observable VOI change in SCA1-affected individuals

**Figure 4:**
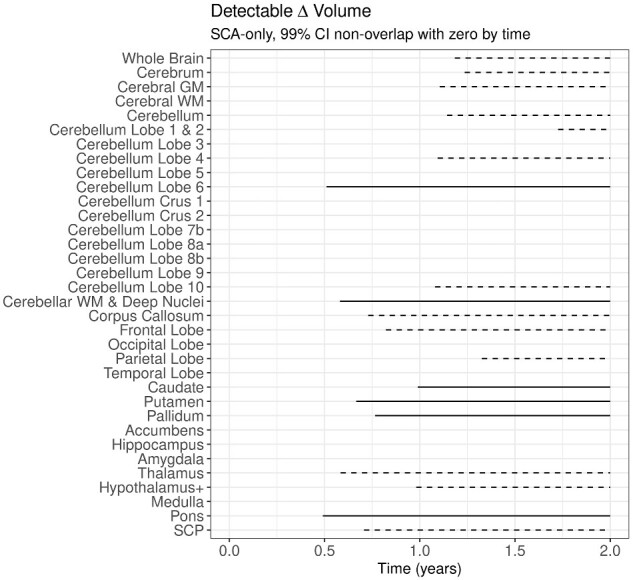
**Timeframe for observable VOI change in SCA1-affected individuals.** Horizontal lines represent the timeframe over which volumetric changes are detectable in SCA1-affected individuals, based on where the 99% confidence interval of change in volume does not overlap with 0 (no change). Solid lines represent VOIs where significant change relative to unaffected individuals was also observed. Dashed lines represent VOIs where SCA1-affected individuals change over time but this change in volume is not different from that observed in unaffected individuals

Potential markers are more useful for evaluation of treatment efficacy if change (or lack thereof) is observable within a short timeframe. To examine when change in each VOI can be reliably detected, we extrapolated from our LME models within SCA1-affected individuals only. Specifically, we calculated the elapsed time at which the 99% confidence interval of the models no longer overlapped zero (i.e. 99% confidence that the change is non-zero). Our models indicated that several VOIs exhibit reliable change within 6–12 months, and critically, these VOIs are the same regions that differentiate SCA1-affected and -unaffected individuals. These VOIs include: cerebellar Lobule 6 (0.51 year), cerebellar WM and deep nuclei (0.58 year), caudate (0.99 year), putamen (0.67 year), pallidum (0.76 year) and pons (0.49 year) (Fig. 4). For elapsed time to detectable differences for all VOIs, see Supplementary Table 4.

### Predicting SCA1 disease progression with VOIs

**Figure 5:**
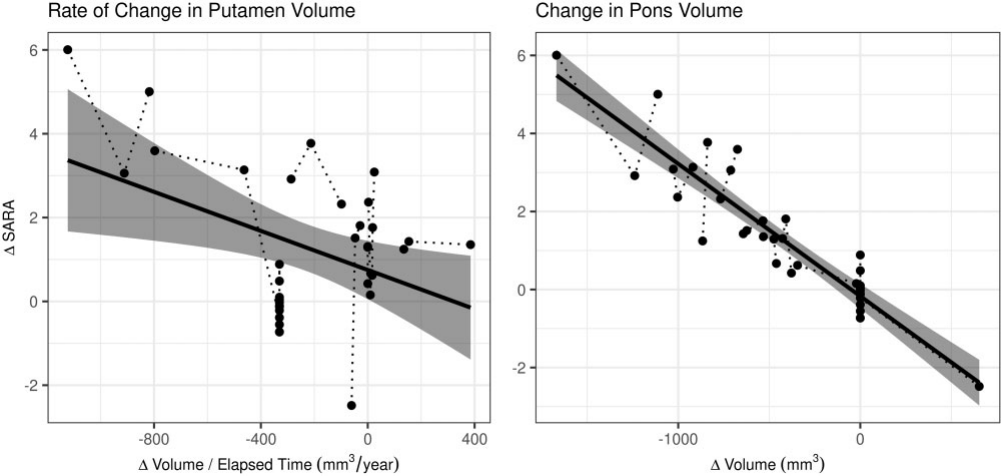
**Predictors of SCA1 disease progression.** The rate of change in putamen volume (left panel) and the change in pontine volume (right panel) predict change in SARA scores. For both of these, VOIs greater (or faster) volume loss resulted in higher SARA scores, which indicates worsening motor symptoms. Circles indicate observed values, where values for individuals are connected by dotted lines. The solid line indicates the fitted model, where the shaded region represents the 95% confidence interval

In relation to our final criterion for a potential biomarker—biomarkers must track disease progression—we assessed which VOIs best predict change in SARA scores using an LME-least absolute shrinkage and selection operator procedure. First, we varied the starting lambda values, which control the penalization of regression parameters from 1 to 100 and compared model fits using Bayesian information criteria. The optimal lambda value is indicated where Bayesian information criteria is least; for our data, the optimal lambda was 37. LME-least absolute shrinkage and selection operator regression indicates that change in pontine volume predicts change in SARA scores [*β* = −0.00272, *z* = –2.235, *P* = 0.0254] (Fig. 5, right panel); this is consistent with previously reported results, which suggested that pontine volume was among the most sensitive markers of disease progression in SCA1-affected individuals (Reetz *et al.*, 2013; Deelchand *et al.*, 2019). In addition, we observe that the *rate of change* in putamen volume predicts change in SARA score, as indicated by a significant putamen by elapsed time interaction [*β* = 0.000419, *z* = −2.393, *P* = 0.0167] (Fig. 5, left panel). All other terms excluding the pontine volume by elapsed time interaction were reduced to zero by the least absolute shrinkage and selection operator regularization procedure but were not significant [*β* = 0.000297, *z* = 0.640, *P* = 0.5222].

## Discussion

Our analyses indicate the *change in pontine volume* and the *rate of change in putamen volume* may provide clinically useful biomarkers to track progression of SCA1. First, they predicted increases in SCA1 disease burden as measured by SARA. Second, change in these volumes was detectable within a short amount of time (∼6–9 months). Third, these changes were observable in SCA1-affected individuals without referencing an unaffected comparison group. Fourth, changes differentiated between SCA1-affected and -unaffected individuals. Finally, both of these measures are obtainable using standard, non-invasive, clinically available MR imaging.

These results are consistent with previous work suggesting that pontine volume is a sensitive potential biomarker in SCA1 (Deelchand *et al.*, 2019) and that decrease in putamen volume is present in SCA1 (Reetz *et al.*, 2013). Our analysis leverages the longitudinal nature of our sample to predict the timeframe in which these changes are detectable, and specifically relate these potential biomarkers to a measure of disease burden (SARA score). From a methodological perspective, this work provides a framework for robustly labelling brain regions in an automated fashion using the MAGMA procedure, which builds upon and optimizes state-of-the-art, multi-atlas labelling techniques. In addition, our data manipulation and modelling procedures provide a robust approach to account for non-linear relationships to ICV, repeated measures in a longitudinal design using a LME framework and a variable selection procedure that can be used when data exhibit multicollinearity and have a potential for over-fitting.

In relation to prior work regarding the pons where both decreasing volume over time and neurochemical abnormalities were identified as important potential biomarkers (Deelchand *et al.*, 2019), it is important to consider how these potential biomarkers may be useful for evaluation of therapeutics. For example, volumetric quantification may prove useful in evaluating the preventative potential of a given treatment, i.e. by virtue of reducing the steepness of the slope of decline or preventing decline altogether. However, volumes are unlikely to increase due to regeneration of neuronal populations in regions where neurons have been lost. By contrast, regional metabolite abnormalities, while less sensitive to disease progression relative to volumetrics (Deelchand *et al.*, 2019), may be reversible and thereby provide insight into functional normalization of already damaged tissues where volumetric change may be obscured.

Our data suggest that cerebellar volume is not an adequate biomarker for disease progression after motor onset in SCA1. While the primary neuronal pathology is the loss of Purkinje cells in the cerebellum (Dürr *et al.*, 1996; Lin *et al.*, 2000; Edamakanti *et al.*, 2018), this may occur long before motor onset. This is supported by our study and the Reetz study in that at the baseline assessment, the volume of the cerebellum was already quite low. On the other hand, the pons was also very low at baseline and did show substantial change over time. Although there was significant change over time in sub-regions of the cerebellum (Lobe 6 and WM/deep nuclei), these changes were not directly related to changes in motor score (SARA).

Lack of relationship between change of cerebellar structure and symptoms highlight the need to move beyond evaluation of regions of interest and consider circuitry. Our results are consistent with the notion that compensatory mechanisms within cerebellar-connected networks might explain the observed decoupling of the primary pathology of SCA1 (cerebellar atrophy) (Klockgether *et al.*, 1998; Schulz *et al.*, 2010; Jacobi *et al.*, 2012) and progression of ataxia. There are well established striato-cerebellar circuits involved in motor control (Hoshi *et al.*, 2005; Bostan *et al.*, 2010, 2013), and the pathways connecting the cerebellum and putamen in this network relay through the pons. Like the Reetz *et al.* paper, in the current study, the putamen was normal in volume at the time of first assessment, but then had a rapid decline in volume. In addition, in the current study, we found that decline in volume of the putamen to be directly related to a decline in motor function. Although some regions of the cerebellum did continue to decrease over time, there was no association between cerebellar volume decrease and motor function deterioration. Given the integration of the cerebellar–striatal circuitry, we hypothesize that the putamen may potentially play a compensatory role in the pre-manifest and early course of the disease. However, this increases the processing burden on the putamen, and this compensation eventually results in a decrease in putamen function. Consistent with our data, a decline in these compensatory mechanisms in the putamen should then be closely related to disease progression. In addition, since the connections for this network run through and connect to pontine areas, it may be that the decay of this pathway and/or compensation by pontine areas will also contribute to compensation and disease progression when these mechanisms fail. These findings are similar to our work in Huntington’s disease where we find that in the pre-symptomatic phase of the disease, striatal degeneration occurs decades prior to motor onset (van Der Plas *et al*., 2019), and at the same time, there is hyper-connectivity of the cerebellar–putamen circuits as seen using resting state MRI (Tereshchenko *et al*., 2020). Further research, particularly prospective research prior to onset of the primary pathology of SCA1 might help disentangle this potential for compensatory mechanisms.

Given the supposition that the striato-cerebellar motor control networks are impacted by SCA1, future research focussing on functional connectivity and physical connectivity within this network will undoubtedly shed light on the mechanisms of SCA1 disease processes and may point to even stronger potential biomarkers that would be useful in the development of clinical trials. In addition, given that the pons is composed of several nuclei as well as has many WM tracts coursing to and through it, higher resolution neuroimaging and histology of this region would aid in isolating the dysfunctional mechanisms that are associated with SCA1. Currently, it is unclear if this effect is due to a loss of pontine neurons or the loss of WM pathways connecting cerebellar regions to the rest of the brain.

Finally, this is the first study to detect changes in brain structures within a relatively short period of time. Although the follow-up time in the current study was, on average, 1.5 years, our statistical analysis allowed for extrapolation of the model to detect significant changes within the 6–12-month time frame, including the putamen and pons. This is an important finding in the context of utilization of these biomarkers in clinical trials where shorter observation times are highly desired. Further research is needed to address issues surrounding the limitations in temporal fidelity (i.e. short follow-up periods would be ideal to reduce the need for extrapolation to shorter timescales), sample size and the single centre nature of the current study, and while MRI data acquisition is highly translatable the sophisticated analytical methods in regard to volumetry and statistical analyses may require optimization to be more useful in a clinical setting.

In conclusion, we have demonstrated that volumetric MR imaging, particularly within the pons and putamen, might make potent biomarkers for clinical trials of treatments for SCA1. The measurement and modelling procedures utilized here can be readily translated to a clinical setting and provide a framework to study the potential for isolating similar mechanisms as useful biomarkers across the broader family of spinocerebellar ataxias.

## Supplementary material

Supplementary material is available at *Brain Communications* online.

## Funding

This work was supported by the National Institute of Neurological Disorders and Stroke (NINDS) (grants R01 NS070815 and R01 NS080816) and Jay D. Schlueter Ataxia Research Fund. The Center for Magnetic Resonance Research is supported by the National Institute of Biomedical Imaging and Bioengineering (NIBIB) (grant P41 EB015894) and the Institutional Center Cores for Advanced Neuroimaging (award P30 NS076408). Research reported in this publication was also supported by the National Center for Advancing Translational Sciences of the National Institutes of Health (award UL1TR000114). The content is solely the responsibility of the authors and does not necessarily represent the official views of the National Institutes of Health.

## Competing interests

The authors report no competing interests.

## Supplementary Material

fcaa184_Supplementary_Data

## Abbreviations

ICV =: intracranial volume
LME =: linear mixed effects
MAGMA =: multiple, automatically generated, morphologically matched atlas approach
MR =: magnetic resonance
SARA =: Scale for the Assessment and Rating of Ataxia
SCA =: spinocerebellar ataxia
SCA1 =: spinocerebellar ataxia Type 1
SCA3 =: spinocerebellar ataxia Type 3
SCA6 =: spinocerebellar ataxia Type 6
WM =: white matter
VOI =: volume of interest
