# Supplementary material for: Brainstem and striatal volume changes are detectable in under 1 year and predict motor decline in spinocerebellar ataxia type 1
Source: Brain Commun. 2020 Dec 15;2(2):fcaa184. doi: 10.1093/braincomms/fcaa184 (PMC7772094; doi:10.1093/braincomms/fcaa184)

## Supplementary Table 1. Relationships between VOIs and ICV

| VOI | Correlation with ICV,  before correction | Estimated Scaling parameter, 𝛼 | Estimated power parameter, 𝛽 | Correlation with ICV, after power proportion |
| --- | --- | --- | --- | --- |
| Whole Brain | 0.947 | 0.115 | 1.14 | -0.031 |
| Cerebrum | 0.949 | 0.0998 | 1.14 | -0.0279 |
| Cerebral GM | 0.908 | 0.0259 | 1.19 | -0.0268 |
| Cerebral WM | 0.901 | 1.13E-01 | 1.07 | -0.0107 |
| Cerebellum | 0.581 | 0.103 | 0.98 | -0.0124 |
| Cerebellar Lobule 1 & 2 | 0.434 | 2.81E-04 | 0.934 | -0.0176 |
| Cerebellar Lobule 3 | 0.516 | 4.3E-05 | 1.23 | -0.000471 |
| Cerebellar Lobule 4 | 0.509 | 0.00154 | 1.04 | -0.0246 |
| Cerebellar Lobule 5 | 0.528 | 0.00696 | 0.978 | -0.00592 |
| Cerebellar Lobule 6 | 0.37 | 0.779 | 0.698 | -0.00697 |
| Cerebellum Crus 1 | 0.375 | 0.959 | 0.712 | 0.00354 |
| Cerebellum Crus 2 | 0.391 | 0.000657 | 1.2 | -0.0197 |
| Cerebellar Lobule 7b | 0.572 | 0.000261 | 1.21 | -0.0366 |
| Cerebellar Lobule 8a | 0.449 | 0.00228 | 1.07 | -0.00423 |
| Cerebellar Lobule 8b | 0.433 | 0.00606 | 0.979 | -0.0061 |
| Cerebellar Lobule 9 | 0.484 | 0.01 | 0.947 | 0.00164 |
| Cerebellar Lobule 10 | 0.363 | 0.163 | 0.63 | 0.00668 |
| Cerebellar WM & Deep Nuclei | 0.473 | 0.000107 | 1.32 | -0.0187 |
| Corpus Callosum | 0.219 | 11.3 | 0.382 | -0.00899 |
| Frontal Lobe | 0.904 | 0.0197 | 1.19 | -0.032 |
| Occipital Lobe | 0.787 | 0.138 | 0.96 | 0.000923 |
| Parietal Lobe | 0.918 | 0.0952 | 1.04 | -0.00941 |
| Temporal Lobe | 0.904 | 0.002 | 1.29 | -0.0196 |
| Caudate | 0.598 | 0.00305 | 1.02 | 0.0205 |
| Putamen | 0.575 | 0.0294 | 0.883 | 0.00294 |
| Pallidum | 0.559 | 0.0629 | 0.747 | 0.0135 |
| Accumbens | 0.769 | 0.00756 | 0.976 | 0.0234 |
| Hippocampus | 0.804 | 1.99E-05 | 1.3 | 0.00489 |
| Amygdala | 0.557 | 2.51E-06 | 1.38 | -0.0375 |
| Thalamus | 0.771 | 0.0598 | 0.877 | -0.0203 |
| Hypothalamus+ | 0.827 | 0.00168 | 1.09 | -0.00643 |
| Medulla | 0.631 | 1.55E-05 | 1.37 | 0.0114 |
| Pons | 0.593 | 8.88E-08 | 1.81 | -0.00416 |
| Superior Cerebellar Peduncle | 0.479 | 3.19E-09 | 1.75 | 0.000392 |

## Supplementary Table 2. Group differences full results

| ROI | Group | Elapsed Time | Baseline Age | Gender | Group by Elapsed Time |
| --- | --- | --- | --- | --- | --- |
| Whole Brain | B=-1.73e+03,  SE=1.5e+04,  t(64.6)=-0.115,  p=0.909 | B=-4.15e+03,  SE=4.42e+03,  t(61.1)=-0.94,  p=0.351 | B=-449,  SE=431,  t(32.5)=-1.04,  p=0.305 | B=-6.18e+03,  SE=1.19e+04,  t(30.9)=-0.519,  p=0.608 | B=-8.42e+03,  SE=6.62e+03,  t(60.3)=-1.27,  p=0.209 |
| Cerebrum | B=-2.4e+03, SE=1.46e+04, t(63.8)=-0.164, p=0.87 | B=-3.95e+03, SE=4.27e+03, t(61.2)=-0.924, p=0.359 | B=-425, SE=423, t(32.6)=-1, p=0.323 | B=-4.85e+03, SE=1.17e+04, t(31)=-0.414, p=0.682 | B=-6.86e+03, SE=6.4e+03, t(60.3)=-1.07, p=0.288 |
| Cerebral GM | B=-2.14e+03, SE=8.71e+03, t(60.6)=-0.245, p=0.807 | B=-1.19e+03, SE=2.46e+03, t(60.7)=-0.484, p=0.63 | B=-69.3, SE=257, t(32.2)=-0.27, p=0.789 | B=-3.08e+03, SE=7.11e+03, t(30.8)=-0.433, p=0.668 | B=-5.33e+03, SE=3.68e+03, t(59.9)=-1.45, p=0.153 |
| Cerebral WM | B=332, SE=4.86e+03, t(63.8)=0.0682, p=0.946 | B=-1.69e+03, SE=1.41e+03, t(61.6)=-1.19, p=0.237 | B=-253, SE=141, t(33.1)=-1.8, p=0.0815 | B=-519, SE=3.9e+03, t(31.5)=-0.133, p=0.895 | B=82.6, SE=2.12e+03, t(60.7)=0.039, p=0.969 |
| Cerebellum | B=273, SE=837, t(57.3)=0.326, p=0.745 | B=138, SE=225, t(60.6)=0.612, p=0.543 | B=-11.2, SE=25.2, t(32.3)=-0.446, p=0.659 | B=-350, SE=699, t(31)=-0.501, p=0.62 | B=-1.11e+03, SE=337, t(60)=-3.3, p=0.00165 |
| Cerebellar Lobule 1 & 2 | B=-0.0419, SE=4.63, t(68)=-0.00905, p=0.993 | B=-4.52, SE=1.42, t(61.2)=-3.19, p=0.00222 | B=-0.223, SE=0.13, t(32.4)=-1.72, p=0.0957 | B=4.8, SE=3.59, t(30.6)=1.34, p=0.191 | B=6.18, SE=2.13, t(60.3)=2.91, p=0.00508 |
| Cerebellar Lobule 3 | B=5.46, SE=25.7, t(59.8)=0.212, p=0.833 | B=8.13, SE=7.15, t(60.9)=1.14, p=0.26 | B=1.07, SE=0.763, t(32.5)=1.41, p=0.169 | B=-29.3, SE=21.1, t(31.1)=-1.39, p=0.176 | B=-3.67, SE=10.7, t(60.2)=-0.343, p=0.733 |
| Cerebellar Lobule 4 | B=-10.3, SE=46, t(62.8)=-0.223, p=0.824 | B=-12.6, SE=13.2, t(61.2)=-0.95, p=0.346 | B=-1.98, SE=1.34, t(32.7)=-1.48, p=0.149 | B=-1.73, SE=37.1, t(31.2)=-0.0467, p=0.963 | B=-14.1, SE=19.9, t(60.4)=-0.709, p=0.481 |
| Cerebellar Lobule 5 | B=4.6, SE=91.2, t(64.2)=0.0504, p=0.96 | B=63.4, SE=27.1, t(59.7)=2.33, p=0.0229 | B=-2.08, SE=2.61, t(30.9)=-0.798, p=0.431 | B=50.2, SE=72.1, t(29.3)=0.696, p=0.492 | B=-105, SE=40.7, t(58.8)=-2.57, p=0.0126 |
| Cerebellar Lobule 6 | B=-25.9, SE=95.3, t(62.8)=-0.271, p=0.787 | B=-19.5, SE=27.5, t(61.1)=-0.711, p=0.48 | B=0.949, SE=2.77, t(32.6)=0.342, p=0.735 | B=22.7, SE=76.8, t(31)=0.295, p=0.77 | B=-191, SE=41.2, t(60.3)=-4.64, p=1.91e-05 |
| Cerebellum Crus 1 | B=29.1, SE=272, t(66.1)=0.107, p=0.915 | B=84.7, SE=81.3, t(61.3)=1.04, p=0.302 | B=1.86, SE=7.74, t(32.7)=0.24, p=0.812 | B=67.5, SE=214, t(31)=0.316, p=0.754 | B=-213, SE=122, t(60.4)=-1.75, p=0.0859 |
| Cerebellum Crus 2 | B=-84.5, SE=413, t(73.1)=-0.205, p=0.838 | B=202, SE=131, t(63)=1.54, p=0.129 | B=-21.1, SE=11.3, t(34.3)=-1.88, p=0.0687 | B=52.8, SE=310, t(32.2)=0.17, p=0.866 | B=-398, SE=197, t(61.9)=-2.02, p=0.0481 |
| Cerebellar Lobule 7b | B=177, SE=213, t(76.4)=0.829, p=0.41 | B=19.1, SE=70.6, t(62.3)=0.271, p=0.787 | B=13.9, SE=5.62, t(33.1)=2.48, p=0.0184 | B=-693, SE=154, t(30.8)=-4.49, p=9.4e-05 | B=-5.1, SE=106, t(61)=-0.0481, p=0.962 |
| Cerebellar Lobule 8a | B=42.5, SE=204, t(66.7)=0.209, p=0.835 | B=-137, SE=61.1, t(61.8)=-2.24, p=0.0288 | B=3.21, SE=5.8, t(33.2)=0.554, p=0.583 | B=-44, SE=160, t(31.5)=-0.275, p=0.785 | B=154, SE=91.6, t(60.9)=1.68, p=0.0973 |
| Cerebellar Lobule 8b | B=71.8, SE=145, t(61.2)=0.494, p=0.623 | B=-76.5, SE=41, t(61.2)=-1.87, p=0.0667 | B=-0.204, SE=4.28, t(32.7)=-0.0477, p=0.962 | B=24.5, SE=119, t(31.3)=0.207, p=0.838 | B=57.5, SE=61.4, t(60.4)=0.936, p=0.353 |
| Cerebellar Lobule 9 | B=41.5, SE=91.8, t(66.5)=0.452, p=0.653 | B=38, SE=27.8, t(60.4)=1.37, p=0.177 | B=-1.35, SE=2.59, t(31.6)=-0.519, p=0.607 | B=14.3, SE=71.6, t(29.8)=0.2, p=0.843 | B=-45.3, SE=41.8, t(59.4)=-1.09, p=0.282 |
| Cerebellar Lobule 10 | B=19, SE=44.4, t(56.7)=0.427, p=0.671 | B=3.98, SE=11.9, t(60.5)=0.335, p=0.739 | B=-0.587, SE=1.34, t(32.1)=-0.439, p=0.664 | B=-22, SE=37.1, t(30.8)=-0.594, p=0.557 | B=28, SE=17.8, t(59.8)=1.58, p=0.12 |
| Cerebellar WM & Deep Nuclei | B=-15.5, SE=133, t(61.3)=-0.116, p=0.908 | B=-49.4, SE=37.6, t(61.2)=-1.31, p=0.194 | B=-2.13, SE=3.91, t(32.8)=-0.544, p=0.59 | B=172, SE=108, t(31.3)=1.58, p=0.123 | B=-259, SE=56.3, t(60.5)=-4.59, p=2.28e-05 |
| Corpus Callosum | B=-0.553, SE=38.5, t(57.2)=-0.0143, p=0.989 | B=-19.1, SE=10.3, t(60.8)=-1.85, p=0.0692 | B=0.816, SE=1.16, t(32.5)=0.702, p=0.488 | B=-13.3, SE=32.2, t(31.2)=-0.414, p=0.682 | B=-26.9, SE=15.5, t(60.2)=-1.74, p=0.0875 |
| Frontal Lobe | B=-799, SE=6.72e+03, t(58)=-0.119, p=0.906 | B=94, SE=1.82e+03, t(60.8)=0.0516, p=0.959 | B=-126, SE=202, t(32.4)=-0.627, p=0.535 | B=-1.18e+03, SE=5.59e+03, t(31.1)=-0.211, p=0.834 | B=-5.56e+03, SE=2.73e+03, t(60.1)=-2.04, p=0.0459 |
| Occipital Lobe | B=-258, SE=1.85e+03, t(57)=-0.14, p=0.889 | B=-803, SE=494, t(60.7)=-1.63, p=0.109 | B=17.2, SE=55.7, t(32.3)=0.309, p=0.759 | B=-460, SE=1.55e+03, t(31.1)=-0.298, p=0.768 | B=521, SE=740, t(60)=0.705, p=0.484 |
| Parietal Lobe | B=-621, SE=4.35e+03, t(56.8)=-0.143, p=0.887 | B=-1.81e+03, SE=1.16e+03, t(60.5)=-1.56, p=0.124 | B=-98.8, SE=131, t(32.1)=-0.754, p=0.456 | B=953, SE=3.64e+03, t(30.8)=0.262, p=0.795 | B=-480, SE=1.74e+03, t(59.8)=-0.275, p=0.784 |
| Temporal Lobe | B=-290, SE=2.51e+03, t(63.2)=-0.115, p=0.908 | B=230, SE=729, t(60.9)=0.316, p=0.753 | B=-129, SE=72.8, t(32.3)=-1.77, p=0.0858 | B=-1.69e+03, SE=2.01e+03, t(30.7)=-0.84, p=0.407 | B=1.11e+03, SE=1.09e+03, t(60.1)=1.01, p=0.315 |
| Caudate | B=43.3, SE=59.9, t(62.4)=0.724, p=0.472 | B=-0.811, SE=17.3, t(60.6)=-0.0469, p=0.963 | B=1.36, SE=1.74, t(32)=0.779, p=0.442 | B=-79.4, SE=48.2, t(30.4)=-1.65, p=0.11 | B=-106, SE=25.9, t(59.8)=-4.1, p=0.000127 |
| Putamen | B=22.1, SE=64.9, t(63.5)=0.341, p=0.734 | B=-4.47, SE=19.1, t(60)=-0.234, p=0.816 | B=-0.454, SE=1.87, t(31.3)=-0.243, p=0.81 | B=-85.5, SE=51.6, t(29.7)=-1.66, p=0.108 | B=-137, SE=28.7, t(59.2)=-4.77, p=1.22e-05 |
| Pallidum | B=11.7, SE=33.7, t(59.7)=0.346, p=0.73 | B=17, SE=9.36, t(60.8)=1.82, p=0.0736 | B=-0.359, SE=0.999, t(32.3)=-0.36, p=0.721 | B=-30.7, SE=27.7, t(30.9)=-1.11, p=0.276 | B=-75.5, SE=14, t(60)=-5.38, p=1.3e-06 |
| Accumbens | B=16, SE=73.7, t(62.3)=0.218, p=0.828 | B=-9.81, SE=21.1, t(61.2)=-0.464, p=0.644 | B=-2.55, SE=2.15, t(32.7)=-1.19, p=0.244 | B=-93.7, SE=59.6, t(31.1)=-1.57, p=0.126 | B=12.3, SE=31.7, t(60.4)=0.39, p=0.698 |
| Hippocampus | B=1.37, SE=24.2, t(69.1)=0.0567, p=0.955 | B=5.84, SE=7.57, t(59.8)=0.772, p=0.443 | B=-0.873, SE=0.669, t(30.8)=-1.31, p=0.201 | B=-7.94, SE=18.4, t(28.9)=-0.431, p=0.67 | B=-4.38, SE=11.4, t(58.8)=-0.385, p=0.702 |
| Amygdala | B=4.01, SE=16.2, t(65)=0.247, p=0.806 | B=-0.689, SE=4.83, t(60.7)=-0.143, p=0.887 | B=-0.58, SE=0.465, t(32)=-1.25, p=0.221 | B=-5.51, SE=12.8, t(30.3)=-0.429, p=0.671 | B=-8.45, SE=7.25, t(59.8)=-1.17, p=0.248 |
| Thalamus | B=60.6, SE=111, t(63.8)=0.546, p=0.587 | B=-151, SE=32.9, t(59.7)=-4.59, p=2.32e-05 | B=2.4, SE=3.18, t(30.9)=0.755, p=0.456 | B=-217, SE=87.9, t(29.3)=-2.47, p=0.0194 | B=-59.8, SE=49.3, t(58.8)=-1.21, p=0.23 |
| Hypothalamus+ | B=-0.36, SE=94.3, t(64.6)=-0.00382, p=0.997 | B=-11, SE=27.7, t(61.3)=-0.395, p=0.694 | B=-3.18, SE=2.72, t(32.8)=-1.17, p=0.249 | B=-34.5, SE=75.1, t(31.1)=-0.459, p=0.649 | B=-78.3, SE=41.6, t(60.5)=-1.88, p=0.0646 |
| Medulla | B=-6.76, SE=147, t(56.2)=-0.046, p=0.963 | B=40, SE=38.9, t(60.6)=1.03, p=0.308 | B=0.851, SE=4.45, t(32.2)=0.191, p=0.85 | B=-74, SE=123, t(31)=-0.599, p=0.553 | B=-29, SE=58.3, t(59.9)=-0.498, p=0.62 |
| Pons | B=37.9, SE=88.8, t(67.6)=0.427, p=0.671 | B=43.4, SE=27.2, t(60.8)=1.6, p=0.115 | B=-0.307, SE=2.49, t(32)=-0.123, p=0.903 | B=-42.3, SE=68.8, t(30.2)=-0.615, p=0.543 | B=-348, SE=40.7, t(59.9)=-8.54, p=5.91e-12 |
| SCP | B=-1.87, SE=6.39, t(63.5)=-0.293, p=0.77 | B=-3.71, SE=1.85, t(61.6)=-2.01, p=0.0492 | B=0.0199, SE=0.186, t(33.1)=0.107, p=0.915 | B=6.8, SE=5.14, t(31.6)=1.32, p=0.196 | B=-4.51, SE=2.77, t(60.8)=-1.62, p=0.109 |

## Supplementary Table 3. Changes in VOI Full LME results

| ROI | Elapsed Time | Baseline Age | Gender |
| --- | --- | --- | --- |
| Whole Brain | B=-1.26e+04, SE=6.06e+03, t(24.7)=-2.08, p=0.0483 | B=-1.04e+03, SE=1.77e+03, t(9.96)=-0.589, p=0.569 | B=-1.15e+04, SE=3.5e+04, t(10.2)=-0.328, p=0.75 |
| Cerebrum | B=-1.08e+04, SE=5.86e+03, t(24.7)=-1.85, p=0.0759 | B=-936, SE=1.76e+03, t(9.97)=-0.531, p=0.607 | B=-7.86e+03, SE=3.49e+04, t(10.2)=-0.226, p=0.826 |
| Cerebral GM | B=-6.52e+03, SE=3.31e+03, t(24.7)=-1.97, p=0.0603 | B=-776, SE=1.01e+03, t(9.98)=-0.768, p=0.46 | B=1.75e+03, SE=2e+04, t(10.2)=0.0874, p=0.932 |
| Cerebral WM | B=-1.61e+03, SE=1.92e+03, t(24.7)=-0.841, p=0.408 | B=60.1, SE=572, t(9.97)=0.105, p=0.918 | B=-7.75e+03, SE=1.13e+04, t(10.2)=-0.685, p=0.509 |
| Cerebellum | B=-972, SE=306, t(24.6)=-3.18, p=0.004 | B=-13.1, SE=92.9, t(9.83)=-0.141, p=0.891 | B=-1.17e+03, SE=1.84e+03, t(10)=-0.636, p=0.539 |
| Cerebellar Lobule 1 & 2 | B=1.64, SE=1.28, t(24.7)=1.29, p=0.209 | B=0.11, SE=0.34, t(9.76)=0.323, p=0.754 | B=-5.45, SE=6.74, t(10)=-0.808, p=0.438 |
| Cerebellar Lobule 3 | B=5, SE=7.27, t(24.7)=0.688, p=0.498 | B=4.48, SE=1.71, t(9.62)=2.62, p=0.0263 | B=-92.9, SE=33.9, t(9.92)=-2.74, p=0.0211 |
| Cerebellar Lobule 4 | B=-26.2, SE=12.2, t(24.3)=-2.14, p=0.043 | B=0.694, SE=2.99, t(9.26)=0.232, p=0.821 | B=-59.3, SE=59.3, t(9.54)=-1, p=0.342 |
| Cerebellar Lobule 5 | B=-40.2, SE=32, t(23.1)=-1.26, p=0.221 | B=0.1, SE=7.32, t(8.1)=0.0137, p=0.989 | B=31.2, SE=145, t(8.38)=0.214, p=0.835 |
| Cerebellar Lobule 6 | B=-211, SE=35, t(24.8)=-6.03, p=2.75e-06 | B=-12.9, SE=9.8, t(9.98)=-1.31, p=0.219 | B=234, SE=194, t(10.2)=1.21, p=0.255 |
| Cerebellum Crus 1 | B=-129, SE=101, t(24.4)=-1.28, p=0.211 | B=-27.2, SE=26.7, t(9.49)=-1.02, p=0.334 | B=933, SE=528, t(9.74)=1.77, p=0.109 |
| Cerebellum Crus 2 | B=-194, SE=173, t(24.8)=-1.12, p=0.273 | B=-39.6, SE=45, t(9.89)=-0.881, p=0.399 | B=-117, SE=891, t(10.2)=-0.131, p=0.898 |
| Cerebellar Lobule 7b | B=11.3, SE=70, t(24.7)=0.162, p=0.873 | B=15.2, SE=16.4, t(9.55)=0.926, p=0.377 | B=-846, SE=325, t(9.85)=-2.6, p=0.0266 |
| Cerebellar Lobule 8a | B=19.1, SE=86.5, t(24.6)=0.22, p=0.827 | B=14.4, SE=20.2, t(9.49)=0.714, p=0.493 | B=-571, SE=401, t(9.79)=-1.42, p=0.186 |
| Cerebellar Lobule 8b | B=-18.6, SE=46.3, t(24.8)=-0.402, p=0.691 | B=18.5, SE=12.2, t(9.86)=1.52, p=0.16 | B=-427, SE=242, t(10.1)=-1.77, p=0.107 |
| Cerebellar Lobule 9 | B=-5.62, SE=38.1, t(24.1)=-0.148, p=0.884 | B=6.42, SE=8.24, t(8.84)=0.779, p=0.456 | B=-219, SE=164, t(9.16)=-1.34, p=0.214 |
| Cerebellar Lobule 10 | B=31.7, SE=14.1, t(25)=2.26, p=0.0328 | B=4.03, SE=3.31, t(9.95)=1.22, p=0.251 | B=-182, SE=65.6, t(10.3)=-2.77, p=0.0194 |
| Cerebellar WM & Deep Nuclei | B=-309, SE=46, t(24.7)=-6.71, p=5.18e-07 | B=8.07, SE=12.3, t(9.85)=0.654, p=0.528 | B=122, SE=245, t(10.1)=0.501, p=0.627 |
| Corpus Callosum | B=-46, SE=11.6, t(24.6)=-3.96, p=0.000569 | B=1.14, SE=3.94, t(9.97)=0.289, p=0.778 | B=48.9, SE=77.8, t(10.1)=0.629, p=0.543 |
| Frontal Lobe | B=-5.45e+03, SE=1.64e+03, t(24.9)=-3.33, p=0.00271 | B=-1.9, SE=424, t(9.98)=-0.00449, p=0.997 | B=-5.82e+03, SE=8.41e+03, t(10.2)=-0.692, p=0.504 |
| Occipital Lobe | B=-285, SE=708, t(24.6)=-0.402, p=0.691 | B=-48.8, SE=236, t(9.97)=-0.207, p=0.84 | B=-449, SE=4.66e+03, t(10.2)=-0.0964, p=0.925 |
| Parietal Lobe | B=-2.31e+03, SE=1.29e+03, t(24.7)=-1.79, p=0.0858 | B=-404, SE=406, t(9.98)=-0.995, p=0.343 | B=8.19e+03, SE=8.02e+03, t(10.2)=1.02, p=0.331 |
| Temporal Lobe | B=1.4e+03, SE=992, t(24.6)=1.41, p=0.172 | B=-290, SE=222, t(9.42)=-1.3, p=0.223 | B=-5.92e+03, SE=4.42e+03, t(9.74)=-1.34, p=0.211 |
| Caudate | B=-107, SE=25.6, t(24.8)=-4.17, p=0.000321 | B=9.42, SE=6.55, t(9.83)=1.44, p=0.182 | B=-290, SE=130, t(10.1)=-2.23, p=0.0494 |
| Putamen | B=-141, SE=24.8, t(24.7)=-5.68, p=6.75e-06 | B=2.98, SE=7.35, t(9.94)=0.406, p=0.694 | B=-150, SE=145, t(10.2)=-1.03, p=0.325 |
| Pallidum | B=-58.7, SE=13.1, t(24.6)=-4.49, p=0.000144 | B=2.58, SE=4.04, t(9.84)=0.64, p=0.537 | B=-21.6, SE=79.9, t(10)=-0.271, p=0.792 |
| Accumbens | B=3.97, SE=29.2, t(24.7)=0.136, p=0.893 | B=-3.37, SE=7.06, t(9.66)=-0.477, p=0.644 | B=-260, SE=140, t(9.95)=-1.86, p=0.0932 |
| Hippocampus | B=1.55, SE=9, t(25)=0.172, p=0.865 | B=-1.54, SE=2.06, t(9.81)=-0.751, p=0.47 | B=-15.1, SE=40.8, t(10.1)=-0.37, p=0.719 |
| Amygdala | B=-9.14, SE=7.02, t(24.8)=-1.3, p=0.204 | B=-2.97, SE=1.75, t(9.82)=-1.69, p=0.122 | B=31.7, SE=34.8, t(10.1)=0.909, p=0.384 |
| Thalamus | B=-211, SE=33.1, t(24.9)=-6.36, p=1.2e-06 | B=5.24, SE=8.71, t(9.97)=0.602, p=0.561 | B=-281, SE=173, t(10.2)=-1.63, p=0.134 |
| Hypothalamus+ | B=-89.3, SE=30.4, t(24.7)=-2.94, p=0.00699 | B=-4.46, SE=8.79, t(9.94)=-0.507, p=0.623 | B=86.1, SE=174, t(10.2)=0.495, p=0.631 |
| Medulla | B=11.6, SE=28.2, t(24.3)=0.41, p=0.685 | B=7.08, SE=7.38, t(9.34)=0.96, p=0.361 | B=-308, SE=146, t(9.59)=-2.11, p=0.0626 |
| Pons | B=-305, SE=31.1, t(25.1)=-9.8, p=4.7e-10 | B=18.1, SE=7.11, t(9.92)=2.55, p=0.029 | B=-305, SE=141, t(10.2)=-2.16, p=0.0552 |
| SCP | B=-8.25, SE=2.22, t(24.9)=-3.72, p=0.00102 | B=0.652, SE=0.502, t(9.74)=1.3, p=0.224 | B=3.57, SE=9.97, t(10.1)=0.358, p=0.728 |

## Supplementary Table 4. Elapsed time to detectable differences, all VOIs

| VOI | Elapsed Time (years) |
| --- | --- |
| Whole Brain | 1.2 |
| Cerebrum | 1.2 |
| Cerebral GM | 1.1 |
| Cerebral WM | >2 |
| Cerebellum | 1.1 |
| Cerebellar Lobule 1 & 2 | 1.7 |
| Cerebellar Lobule 3 | >2 |
| Cerebellar Lobule 4 | 1.1 |
| Cerebellar Lobule 5 | >2 |
| Cerebellar Lobule 6 | 0.51 |
| Cerebellum Crus 1 | >2 |
| Cerebellum Crus 2 | >2 |
| Cerebellar Lobule 7b | >2 |
| Cerebellar Lobule 8a | >2 |
| Cerebellar Lobule 8b | >2 |
| Cerebellar Lobule 9 | >2 |
| Cerebellar Lobule 10 | 1.1 |
| Cerebellar WM & Deep Nuclei | 0.58 |
| Corpus Callosum | 0.73 |
| Frontal Lobe | 0.82 |
| Occipital Lobe | >2 |
| Parietal Lobe | 1.3 |
| Temporal Lobe | >2 |
| Caudate | 0.99 |
| Putamen | 0.67 |
| Pallidum | 0.76 |
| Accumbens | >2 |
| Hippocampus | >2 |
| Amygdala | >2 |
| Thalamus | 0.58 |
| Hypothalamus+ | 0.98 |
| Medulla | >2 |
| Pons | 0.49 |
| SCP | 0.71 |

Supplemental Figure – LME-LASSO


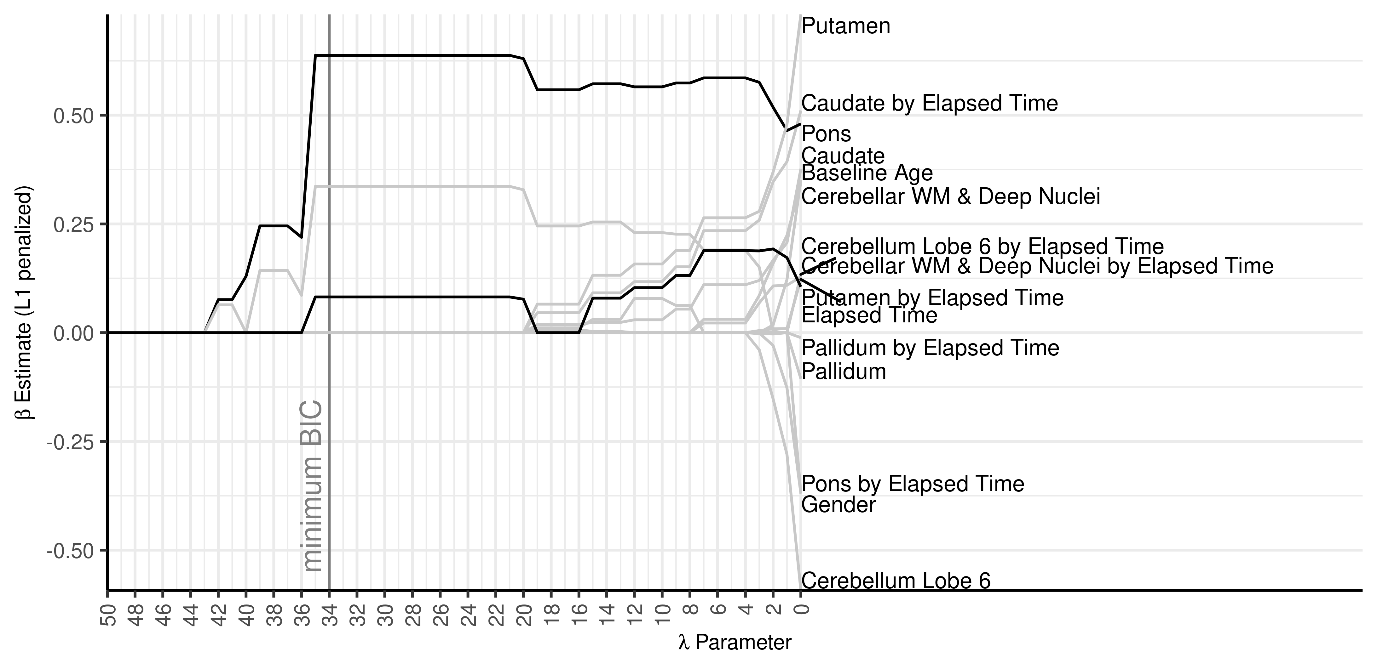

Supplement: fcaa184_Supplementary_Data [file fcaa184_supplementary_data.docx]
